# Antipyrin

**Published:** 1889-07

**Authors:** 


ANTIPYRIN.—The chemical name of this remedy is well known, but as yet no one has ever been known to pronounce it twice the same way. It is *Dimethyloxyquinizine.*

